# Heme arginate improves acute liver failure secondary to erythropoietic protoporphyria: a case report

**DOI:** 10.3389/fmed.2026.1813183

**Published:** 2026-04-23

**Authors:** Hua Ni, Beijin Chen, Aiping Huang, Tian Zhang, Hao Li, Shanhong Tang

**Affiliations:** 1 Department of Gastroenterology, The General Hospital of Western Theater Command, Chengdu, Sichuan, China; 2 Department of Pathology, The General Hospital of Western Theater Command, Chengdu, Sichuan, China; 3 Department of Laboratory and Pathology, Lintong Rehabilitation and Convalescent Center, Xi’an, Shaanxi, China

**Keywords:** abdominal pain, artificial liver support, erythropoietic protoporphyria, heme arginate, jaundice, liver failure

## Abstract

**Introduction:**

Erythropoietic protoporphyria (EPP) is a rare inherited disorder with limited therapeutic options. For EPP patients with advanced cholestasis or liver failure, liver transplantation has long been regarded as the definitive therapy to address the hepatic crisis but does not offer a cure. Previous studies have reported that intravenous hemin/hematin may benefit some EPP patients, while the efficacy of intravenous heme arginate in treating EPP remains unreported.

**Case presentation:**

We report a 24-year-old male with EPP and severe cholestasis. From 2017 to 2024, the patient was hospitalized eight times for recurrent episodes of jaundice and abdominal pain. These symptoms were effectively alleviated through a comprehensive treatment including artificial liver support (PE + DPMAS), hepatoprotective therapy, and high-glucose infusion. During his latest hospitalization in 2025, artificial liver support failed to improve the liver dysfunction, leading to progressive deterioration to liver failure. Notably, after two doses of intravenous heme arginate, the total bilirubin level gradually declined with significant improvement of ascites, coagulation dysfunction and neuropsychiatric symptoms. The patient was discharged with hepatoprotective therapy and light avoidance with no recurrence at 6-month follow-up.

**Conclusion:**

This case suggests that intravenous heme arginate may provide clinical benefit in patients with EPP-associated liver failure and contributes to the limited literature supporting heme-based therapy in treating EPP hepatic crisis.

## Introduction

Erythropoietic protoporphyria (EPP) is characterized by the deficiency of ferrochelatase (FECH), a catalytic enzyme in the final step of heme synthesis (1). Excessive accumulation of protoporphyrin results in hepatocyte and bile duct injury, with the potential to ultimately progress to hepatic failure (2). For EPP patients with advanced cholestasis or cirrhosis, liver transplantation may be a beneficial therapy but does not offer a cure, given that the primary source of excessive protoporphyrin IX remains in the bone marrow (3). Intravenous heme arginate is the specific therapy for acute hepatic porphyrias, namely intermittent porphyria, variegate porphyria, and hereditary coproporphyria (4). Previous studies already suggests that heme-based therapy may benefit some EPP patients, however, the efficacy of intravenous heme arginate in EPP patients has not been reported.

In this case report, we describe a 24-year-old male with EPP and severe cholestasis who was initially successfully managed through a comprehensive treatment including artificial liver support, hepatoprotective therapy, and high-glucose infusion. However, artificial liver support failed to alleviate liver dysfunction in 2025, leading to progressive deterioration to liver failure. Intravenous heme arginate therapy effectively improves fulminant cholestasis of EPP-associated liver failure, suggesting an alternative therapy for addressing EPP-related hepatic crisis.

## Case presentation

**Figure 1:**
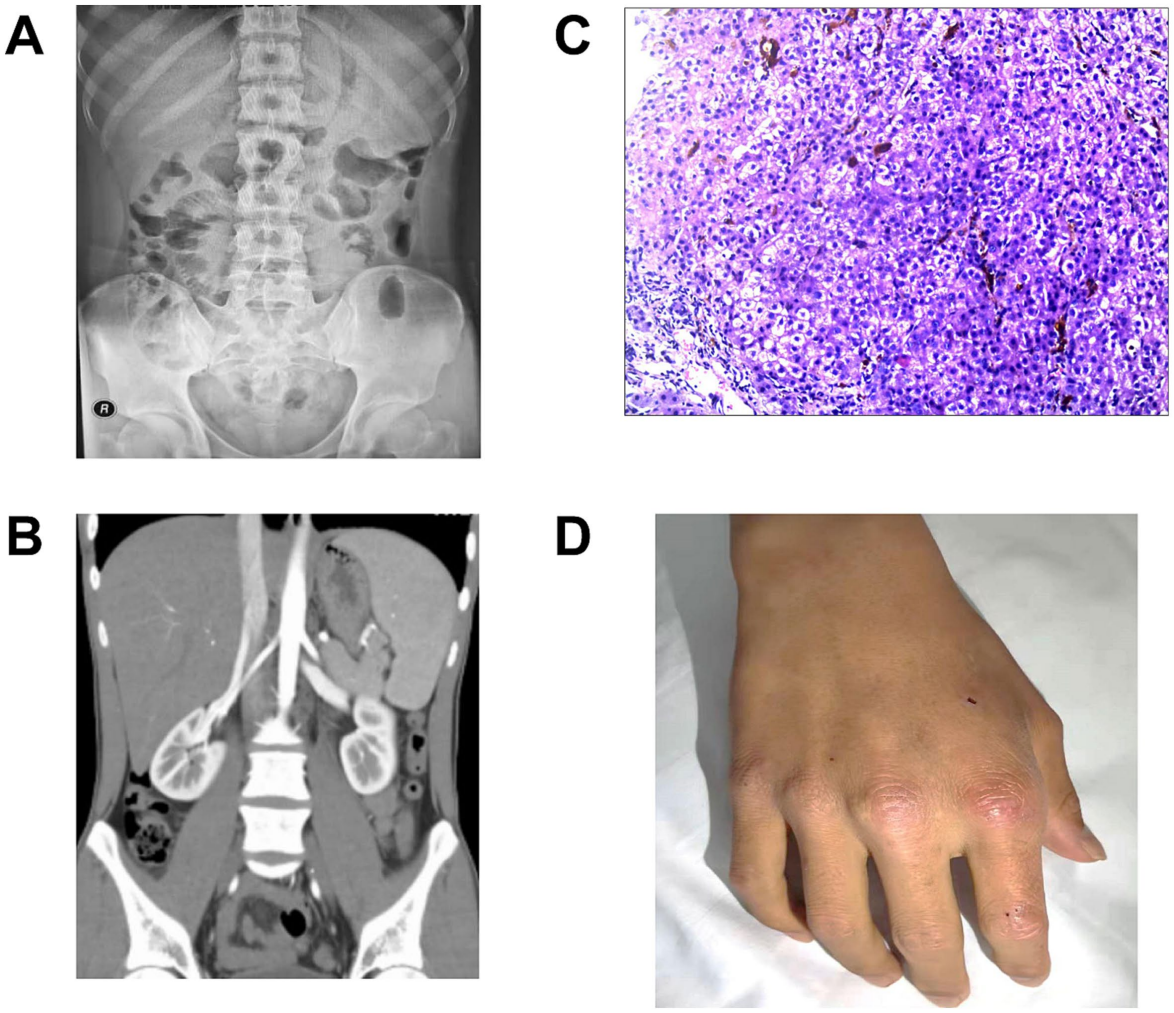
Intestinal obstruction and cholestasis. **(A)** Abdominal X-ray indicated the symptoms of intestinal obstruction in the lower right abdomen. **(B)** Contrast-enhanced abdominal CT indicated hepatosplenomegaly and excluded biliary obstruction, tumors, or portal vascular abnormalities. **(C)** Liver biopsy indicated active cirrhosis and severe intrahepatic cholangitis (HE, ×100). **(D)** Nonblistering cutaneous photosensitivity in light-exposed areas.

**Figure 2:**
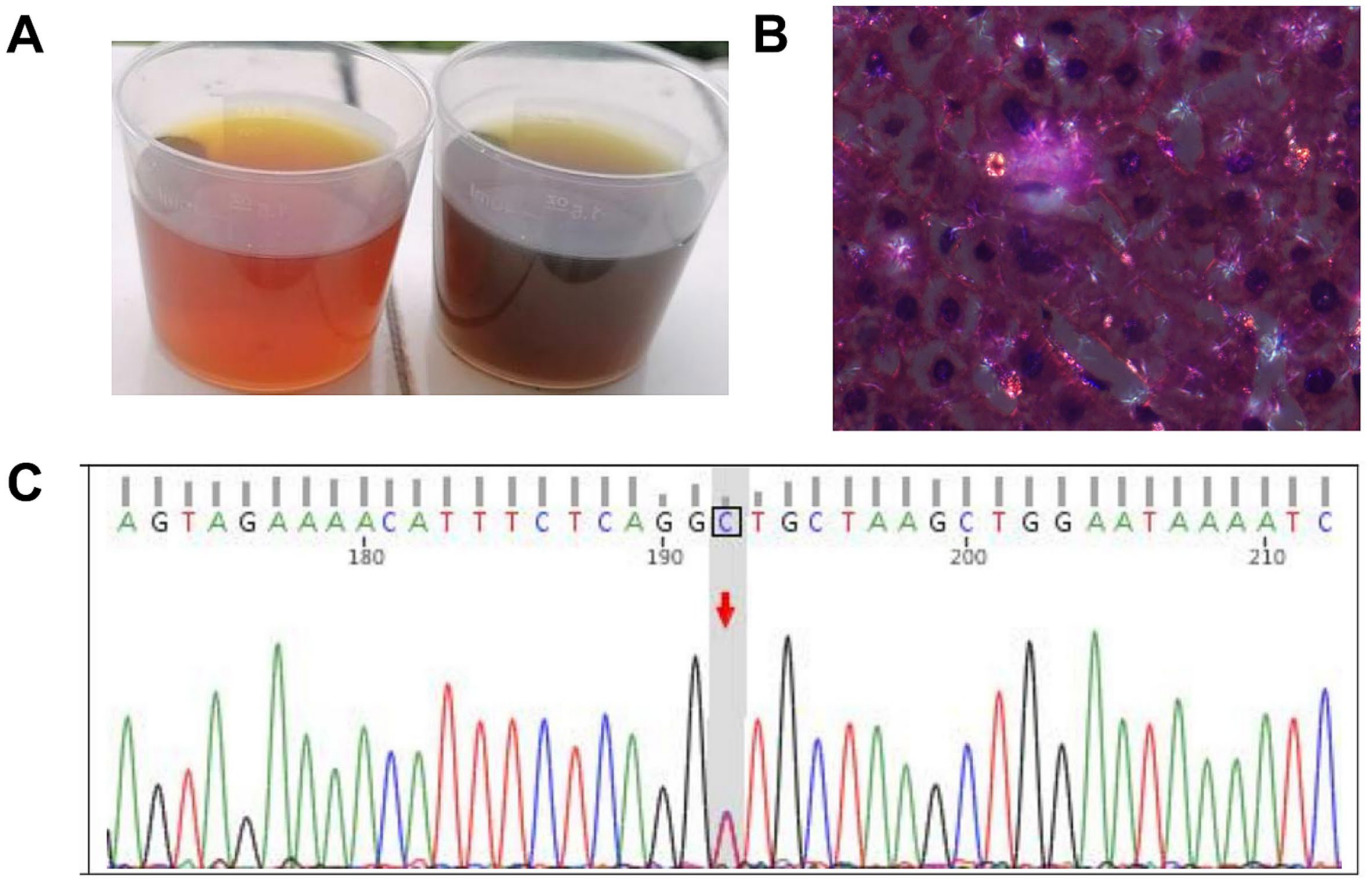
Establishing the diagnosis of EPP. **(A)** The change of urine color after sun exposure indicated the preliminary consideration of EPP. **(B)** Polarized light microscopy indicated the characteristic red birefringence and Maltese cross of EPP (HE, ×400). **(C)** Whole-exome sequencing identified a homozygous splicing mutation (c.315-48 T > C) in the *FECH* gene.

A 24-year-old man presented with a prolonged history of jaundice and abdominal pain lasting over 8 years. Investigations revealed no abnormalities in hepatitis virus series, autoimmune hepatitis antibodies, immunoglobulins, or ceruloplasmin levels. The abdominal X-ray indicated multiple gas–liquid interface in the lower right abdomen (Figure 1A). Contrast-enhanced abdominal computed tomography demonstrated hepatosplenomegaly without evidence of biliary obstruction, tumors, or portal vascular abnormalities (Figure 1B). Liver biopsy indicated nodular hepatocellular hyperplasia and intrahepatic cholangitis (Figure 1C). Based on the patient’s clinical manifestations and history of photosensitivity (Figure 1D), porphyria remained a strong diagnostic consideration. The urine was observed to change from yellow to a brown-red color after sun exposure (Figure 2A). The characteristic findings of red birefringence and Maltese crosses were observed on polarizing microscopy (Figure 2B). Whole-exome sequencing identified a homozygous splicing mutation (c.315-48 T > C) in the FECH gene (Figure 2C). Collectively, these findings established the definitive diagnosis of EPP.

**Figure 3:**
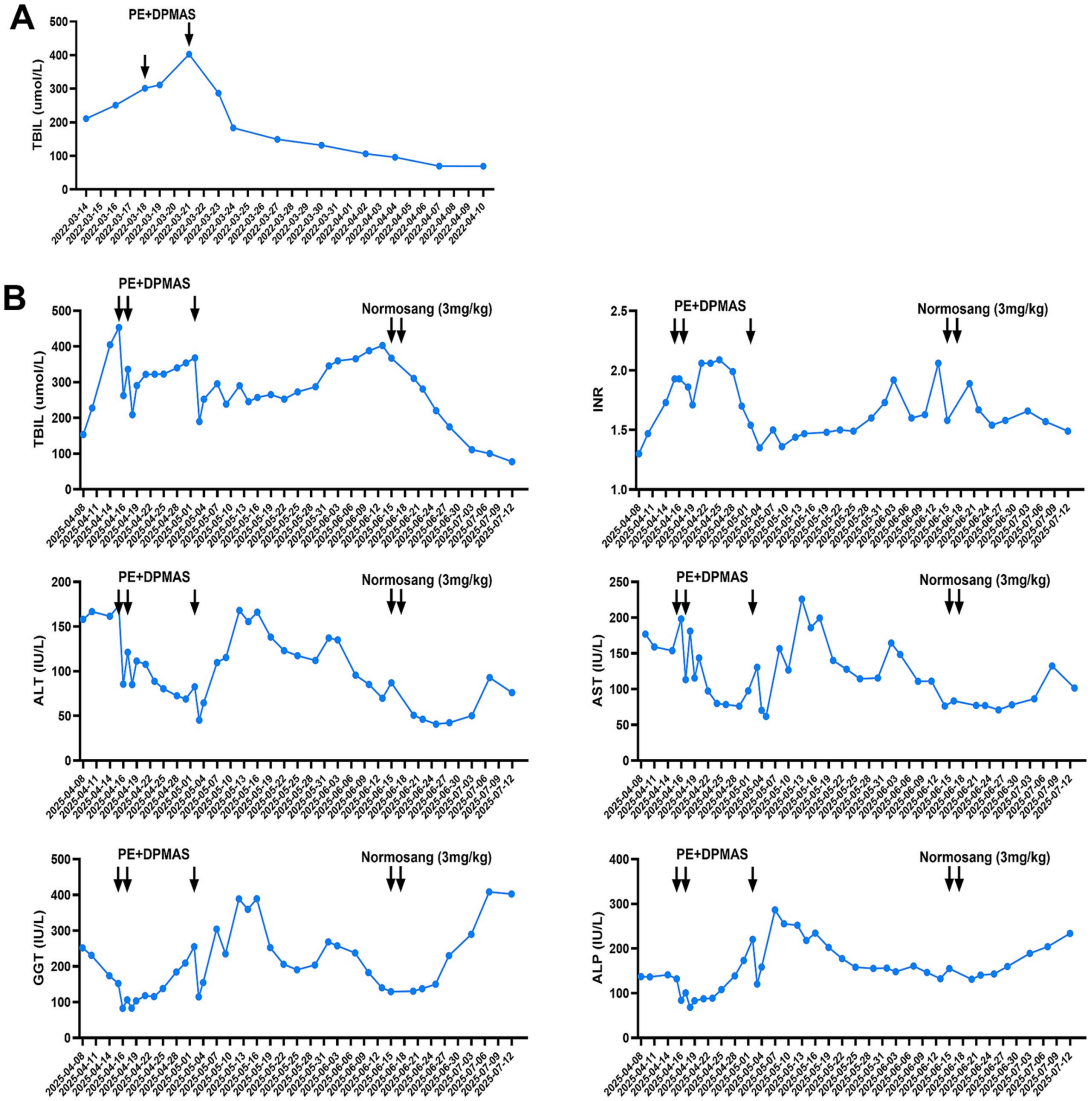
Treatment and follow-up. **(A)** Total bilirubin level was alleviated following comprehensive treatment including artificial liver support (one of the representative hospitalizations in 2022). **(B)** Significant improvement of intravenous heme arginate therapy for EPP-related liver failure after comprehensive treatment including artificial liver support.

From 2017 to 2024, the patient was hospitalized eight times. His jaundice and abdominal pain were alleviated following a course of comprehensive treatment, including artificial liver support (PE + DPMAS), hepatoprotective therapy, and high-glucose infusion (Figure 3A). During his most recent admission in 2025, the total bilirubin (TBIL) rose up to 453.3 μmol/L, international normalized ratio (INR) 1.93, *γ*-glutamyl transpeptidase (GGT) 152.1 IU/L, alkaline phosphatase (ALP) 132.1 IU/L, and alanine aminotransferase (ALT) 173.5 IU/L, aspartate aminotransferase (AST) 198.3 IU/L. However, comprehensive treatment including artificial liver support failed to sequentially improve the liver dysfunction, and his condition progressively deteriorated with the prolonged prothrombin time, ascites, hepatic encephalopathy, and neuropsychiatric symptoms. Notably, after two doses of intravenous heme arginate (Normosang, 3 mg/kg/day), the patient’s total bilirubin level and INR gradually declined (Figure 3B). Ascites and neuropsychiatric symptoms were alleviated without neurological sequelae. The patient was subsequently discharged and advised to continue hepatoprotective therapy and light avoidance. During the six-month follow-up, the patient remained clinically stable and his bilirubin levels were maintained at baseline.

## Discussion

EPP is a syndrome characterized by photosensitivity, abdominal pain, and cholestasis based on the accumulation of protoporphyrin IX in the specific tissues. The symptoms may be nonspecific, so it is difficult to differential diagnosis. Painful nonblistering photosensitivity usually suggests the diagnosis of EPP, we suspected this potential based on further inquiry into the history of cutaneous photosensitivity. As shown in Figure 1D, it represents chronic changes of leathery hyperkeratotic skin and mild scarring especially in hands and finger joints. Patients with severe protoporphyric hepatopathy may develop a severe motor neuropathy similar to acute porphyrias, as characterized by abdominal pain, weakness, constipation, nausea and vomiting (5). In the present case, the initial symptoms were similar with intestinal obstruction, which could be also interpreted by the severe protoporphyric hepatopathy. Protoporphyrin in plasma is taken up by hepatocytes and excreted in bile undergoing enterohepatic circulation. Excess protoporphyrin tends to form crystalline structures in hepatocytes and impair mitochondrial function, leading to cholestasis (6). The patient developed quite severe hepatocellular jaundice, while cholestasis was relatively mild, which may due to the cholestatic protoporphyrin in hepatocyte.

About 5% of EPP patients develop rapid and severe cholestatic hepatitis with liver failure, known as EPP hepatic crisis (7). Previous study reported that the overall mortality rate of EPP was about 4.1% by reviewing 98 cases of EPP reported in the past decade (8). As for genetic diseases, the curable treatment for protoporphyric hepatopathy is limited. Generally, hepatoprotective agents including ursodesoxycholic acid and glutathione were available to alleviate cholestasis and oxidative stress (9). Plasma exchange has been recommended for patients with advanced EPP and liver failure to reduce plasma levels of PPIX and bridge the patient to liver transplant (10). Additionally, liver transplantation may be the only beneficial therapy to address EPP hepatic crisis (11).

Intravenous heme therapy may benefit AIP patients through inhibiting the induction of delta-aminolaevulinic acid synthase 1 (ALAS1), and reducing the formation of potentially harmful metabolites of porphyrin (12). Heme arginate and hemin represents two classical pharmacological options for suppressing ALAS1, both of which have been successfully applied in treating acute attacks of AIP (13). It has been proposed to use intravenous hemin therapy in perioperative management of EPP patients since hemin reduces the protoporphyrin burden (14) and hyperbilirubinemia (15). Bloomer and Pierach reported that IV hematin administered to patients with protoporphyria and decompensated cirrhosis produced biochemical changes consistent with reduced protoporphyrin formation and was proposed as potentially useful (16). Dellon et al. reported that intermittent IV hematin after liver transplantation was associated with remission of recurrent EPP allograft dysfunction for nearly 2 years (17). A 60-year-old man, diagnosed with EPP-related liver failure, achieved temporary benefit but died 2 weeks later despite multidisciplinary treatment including hemin therapy, phlebotomy, and plasma exchange (18). Prior reports have described temporary or supportive benefit from intravenous hemin/hematin in EPP-associated liver disease, but evidence remains sparse, and the role of intravenous heme arginate in this setting has not been clearly established.

The patient previously successfully recovered after comprehensive managements including artificial liver support for several times, while failed to alleviate this time and may deserve liver transplantation to solve such EPP hepatic crisis. Previously, hemin/hematin was reported to be beneficial in treating EPP-related cholestasis/liver failure, we attempted intravenous heme arginate and observed the marked improvement in treating EPP-related liver failure. Intravenous heme therapy is recommended for four times for AIP (4). In the present case, the patients only received two doses due to financial constraints. Intriguingly, the insufficient dose demonstrated a durable and dramatic response in treating EPP-related liver failure. In contrast to hemin, heme arginate therapy indicates several advantages. Hemin solutions are extremely unstable (degradation rates up to 61% in 4 h), resulting in adverse reactions caused by its degradation products (12), while heme arginate is reported to be less side effects and better stability (19). Additionally, hemin is roughly four times more expensive than heme arginate (13). The use of heme arginate specifically rather than hematin/hemin, possibly in native-liver EPP-associated fulminant cholestasis/liver failure, and possibly demonstrating a more durable or dramatic response than prior reports.

However, there are some limitations for this case. The liver biopsy revealed severe intrahepatic cholangitis, yet this finding failed to explain the abdominal pain concomitant with intestinal obstruction. The abdominal pain concomitant with intestinal obstruction may due to the accumulation of protoporphyrin IX in small intestine or colon, which needs the pathological evidence of intestinal tissues. Additionally, the detection of serum protoporphyrin IX is unavailable, failing to provide the direct evidence whether heme arginate can reduce the accumulation of protoporphyrin. Moreover, the family history of inherited nature was incomplete, as we only tested his sister, an asymptomatic carrier of heterozygous mutation in FECH gene (c.315-48 T > C). Given the fact that this is a single case report, it is essential to acknowledge the inherent constraints, including the limited generalizability of the findings, the relatively short follow-up period, and the inability to establish causality. The efficacy and safety of intravenous heme arginate in treating EPP remains to be further investigated in large cohort.

## Conclusion

Current effective therapeutic options for EPP hepatic crisis are limited. Our findings highlight that heme arginate may serve as an alternative therapy for acute hepatic decompensation in EPP.

## Data Availability

The original contributions presented in the study are included in the article/supplementary material, further inquiries can be directed to the corresponding author.
